# Diagnostic Potential of Pulsed Arterial Spin Labeling in Alzheimer's Disease

**DOI:** 10.3389/fnins.2016.00154

**Published:** 2016-04-19

**Authors:** Stefano Trebeschi, Isabelle Riederer, Christine Preibisch, Karl P. Bohn, Stefan Förster, Panagiotis Alexopoulos, Claus Zimmer, Jan S. Kirschke, Alexander Valentinitsch

**Affiliations:** ^1^Department of Neuroradiology, Klinikum rechts der Isar, Technische Universität MünchenMunich, Germany; ^2^Department of Nuclear Medicine, Klinikum rechts der Isar, Technische Universität MünchenMunich, Germany; ^3^Department of Nuclear Medicine, Ulm UniversityUlm, Germany; ^4^TUM Neuroimaging Center, Klinikum rechts der Isar, Technische Universität MünchenMunich, Germany; ^5^Departments of Psychiatry and Psychotherapy, Klinikum rechts der Isar, Technische Universität MünchenMunich, Germany; ^6^Department of Psychiatry, University Hospital of Rion, University of PatrasPatras, Greece

**Keywords:** MRI, PASL, neuroimaging, Alzheimer's disease, diagnosis

## Abstract

Alzheimers disease (AD) is the most common cause of dementia. Although the underlying pathology is still not completely understood, several diagnostic methods are available. Frequently, the most accurate methods are also the most invasive. The present work investigates the diagnostic potential of Pulsed Arterial Spin Labeling (PASL) for AD: a non-invasive, MRI-based technique for the quantification of regional cerebral blood flow (rCBF). In particular, we propose a pilot computer aided diagnostic (CAD) procedure able to discriminate between healthy and diseased subjects, and at the same time, providing visual informative results. This method encompasses the creation of a healthy model, the computation of a voxel-wise likelihood function as comparison between the healthy model and the subject under examination, and the correction of the likelihood function via prior distributions. The discriminant analysis is carried out to maximize the accuracy of the classification. The algorithm has been trained on a dataset of 81 subjects and achieved a sensitivity of 0.750 and a specificity of 0.875. Moreover, in accordance with the current pathological knowledge, the parietal lobe, and limbic system are shown to be the main discriminant factors.

## 1. Introduction

Alzheimers disease (AD) is the most common cause of dementia. Symptoms include memory loss, disturbances in language, psychological and psychiatric changes, and instabilities, as well as impairment of daily life activities. Although, in recent years, many studies reported formation of amyloid-beta peptide plaques in the brain of AD subjects, the underlying cause of AD is still mostly unknown. Moreover, at the present day no efficient treatment is available. Some studies in the past years reported a set of possible protective factors; however, none of them provided consistent and sufficient evidence to be implemented as general health care standard (Burns and Iliffe, 2009; Kawas, 2012; Salloway et al., 2014). The Alzheimers Association reported (Dementia, 2006) the most common problems that early onset AD patients are confronted with: job performance, denied employer assistance, and denied disability status. There are a variety of factors that might lead to the aforementioned consequences, in particular the difficulty of getting an accurate and timely diagnosis because of the unlikelihood of dementia in younger adults.

There are a variety of diagnostic tools and methods being used in clinical as well as medical practice. Most commonly, neuropsychological tests, like the Mini-Mental State Examination (MMSE) (Pasquier, 1999), are used to assess memory disorders. The most accurate method known for the diagnosis of AD relies on the analysis of cerebrospinal fluid, reporting on average a sensitivity >0.80 and a specificity >0.90 (Sjögren et al., 2003). Despite the high accuracy, this method is highly invasive. Neuroimaging techniques are also widely used in the diagnostic process. In the standard diagnostic procedure, structural magnetic resonance imaging (MRI) and functional [18F]-Fluorodeoxyglucose Positron Emission Tomography (FDG-PET) are used for the diagnosis of AD. FDG-PET is by far the best established, most accurate single subject imaging method in the diagnosis of AD. In particular, [18F]-Fluorodeoxyglucose is used to assess abnormalities in the glucose metabolism in the brain. Furthermore, there are specific PET tracers for visualization of amyloid plaques such as [11C]-PiB which showed promising results (Rinne et al., 2010). Currently, there are three officially released amyloid-PET tracers: [18F]-Florbetaben (Piramal), [18F]-Flutemetamol (GE Healthcare), and [18F]-Florbetapir (Eli Lilly/AVID). These PET tracers are currently not broadly used because of either limited distribution and/or restrictions in reimbursement of amyloid PET examination (i.e., in Germany). Structural MRI can be used for the detection of atrophy, in particular of the hippocampus which is characteristic for AD. However, hippocampal atrophy, according to the most accepted hypothetical biomarker model in the pathophysiology of AD, is a structural alteration which usually occurs later than AD-typical FDG-hypometabolic alterations (i.e., in the Precuneus and Posterior Cingulate Cortex). Alternatively, quantification of regional cerebral blood flow (rCBF) is a promising method. rCBF was found to be tightly correlated with the regional consumption of glucose and reflects neuronal activity but SPECT and PET based rCBF measurements also rely on radioactive tracers where a number of restrictions apply (see Chetelat and Baron, 2003; Ewers et al., 2011). A novel, absolutely non-invasive, MR based, imaging technique, called arterial spin-labeling (ASL) might represent a more convenient alternative. ASL is based on the magnetic labeling of arterial water proton spins: inflowing blood is labeled proximal to the tissue of interest, subsequently, brain images are taken once the labeled blood reaches the brain. Quantitative perfusion images are calculated from the difference between labeled and unlabeled images (Detre et al., 1994).

One approach to explore the diagnostic potential of PASL is the formulation of a quantitative diagnostic procedure, which provides, at the same time, anatomical information. Thus, the PASL based perfusion information can be reassured by anatomical information in accordance with current pathophysiological knowledge. Voxel-based morphometry (VBM) is a statistical tool widely used to identify brain volume differences between groups of subjects. At its simplest, this method performs a series of voxel-wise comparisons revealing regions which differ significantly between the two groups (Friston et al., 1995). Although VBM is mostly used for neuroscientific studies, diagnostic procedures based on VBM are not novel in the literature. Most of these methods rely on a custom voxel-wise computation of z-scores between the subject under analysis and a model of the healthy population. A number of successful results based on structural MRI have been reported in Kawachi et al. (2006), Bonilha et al. (2009), Ishii et al. (2005), Colliot et al. (2006), Hirata et al. (2005), and Mühlau et al. (2009). For example, a diagnostic VBM procedure for Glioma detection based on ASL images have been developed (Maumet et al., 2013). More advanced methods relying on VBM have also been proposed, such as classification of features computed on brain regions (Zhang et al., 2008), or fuzzy classifications (Thangaraj et al., 2010). There are several advantages for using VBM. First of all, this method unfolds in a series of comparisons. Compared to more complicated multivariate tools, it is simpler, widely understood and accepted. Moreover, this method benefits from a high level of anatomical information, which is given by the intrinsic spatial information. The localization of significantly different anatomical regions allows to control the quality, meaningfulness, and interpretation of the results.

Our hypothesis is that the diagnosis of AD can be achieved via PASL-based CBF images. In order to prove this, we propose an adapted voxel-based probabilistic comparison method to quantify abnormalities in individual AD patients based only on PASL images. This method provides both quantitative results of a standard computer-aided diagnostic procedure, and high anatomical information of VBM.

## 2. Materials and methods

For the individual diagnosis of AD, we developed an adapted VBM technique for the analysis of rCBF data. This adaptation requires: (1) a CBF model of the healthy population, which will be used as a reference for the comparison with an individual patient (test-subject); (2) a voxel-wise comparison method between the reference model and an individual subject, which will have to classify each voxel of the test-subject as hypo-perfused (i.e., with reduced perfusion) or normal (Alsop et al., 2010). The total number of hypo-perfused voxels will represent the hypo-perfused volume. Our assumption is that a correlation exists between the hypo-perfused volume and the severity of the disease. From this assumption, we strive to determine a discriminant number of hypo-perfused voxels which allows to classify a patient as diseased. Moreover, the method has to take into account the noise of the data and the fact that some hypo-perfusion can be caused by other factors different from AD.

### 2.1. Subjects

We included 81 subjects in the study. In 54 patients and 32 healthy volunteers, a PASL sequence of the brain was acquired. The patients were examined from 2011 to 2014 during their clinical routine examinations for the clarification of their symptoms of dementia. The healthy controls were recruited by announcements in the clinic or relatives of the patients. They had no subjective memory complaints and were independent in their activities of daily living. The healthy controls and patients have all been examined by a psychiatrist, five patients were excluded from the study due to huge motion artifacts. None of the healthy controls had to be excluded. The local institutional review board approved this single-center study. All patients gave informed consent. The study was performed in accordance with the ethical standards of the 1964 Declaration of Helsinki and its later amendments (MAGA, 2004).

Each subject has been classified according to the diagnosis established by a neuropsychiatrist: 32 healthy subjects were included in the healthy cohort (*HC*). The remaining 49 patients were diagnosed with AD: 19 early onset (*AD*_*EA*_) and 30 late onset (*AD*_*LA*_). Additionally, for each subject a set of biomedical and cognitive measurements were acquired: age, gender, MMSE score, and education (in years). We investigated the correlations between the number of hypo-perfused voxels and each biomedical and cognitive measurement.

### 2.2. Image acquisition

**Figure 1:**
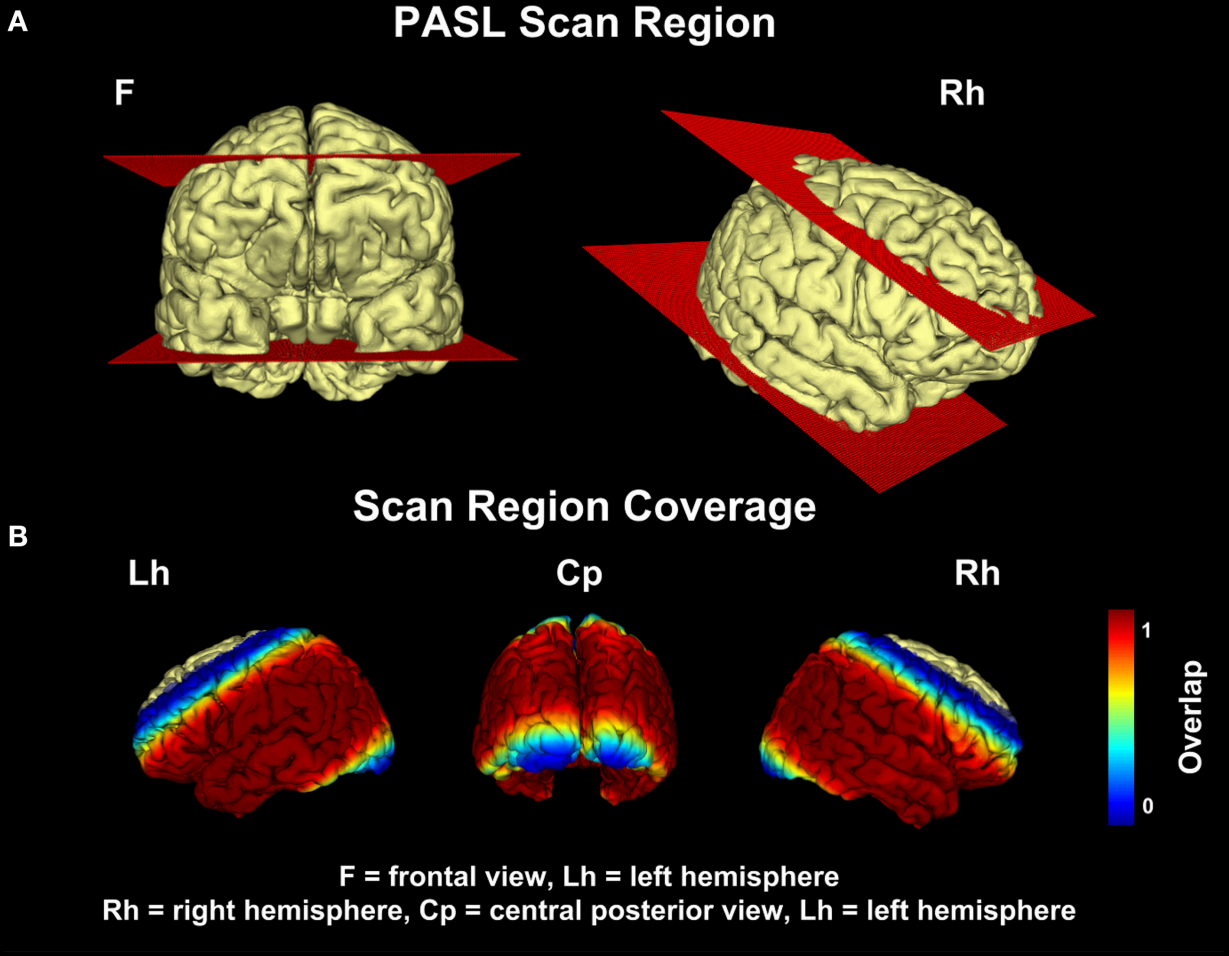
**Pulsed Arterial Spin-Labeling (PASL) scan region. (A)** represents the bounding plane of the PASL signal coverage. **(B)** shows the coverage of the PASL signal throughout the cohort. The intensity values represent the percentage of patients, who showed a non-zero pASL signal at that voxel location. All voxel locations whose coverage was equal or less than 0.95 were discarded.

Image acquisition was performed at 3T either on a Philips Achieva System (Philips Healthcare, Hamburg, Germany) (*n* = 24) or on a Siemens mMR Biograph (Siemens Healthcare, Erlangen, Germany) (*n* = 57). On both scanners, a similar sequence and comparable imaging parameters were used. At the Philips Achieva system, we used the pulsed star labeling of arterial regions (PULSAR) technique (Golay et al., 2005), while the proximal inversion with a control for off-resonance effects (PICORE) labeling technique (Wong et al., 1999) was used at the Siemens mMR Biograph. In both cases single shot EPI was used for image readout with TR = 2500 ms, α = 90° and minimum TE [TE = 17 ms (Philips); TE = 13 ms (Siemens)]. In both cases thin slice periodic saturation pulses were used to obtain a defined bolus (Q2TIPS) (Luh et al., 1999) using TI1, TI1S, TI2 = (700, 1200, 1500 ms). A set of eleven slices [matrix size 64 × 63, voxel size of 3.75 × 3.75 × 6 mm (Philips) or 4 × 4 × 6 mm (Siemens), 0.6 mm gap] aligned to the hippocampus and containing the parietal lobe were acquired in ascending order (see Figure 1). Each measurement comprised eighty pairs of label-control acquisitions. The total scan time was ~440 s. In order to perform a proper alignment between perfusion and structural images, we acquired an EPI volume covering the whole brain with the same voxel sizes in 40 slices, and a T1-weighted Turbo Field Echo sequence with voxel size 1 × 1 × 1 mm in 170 sagittal slices.

### 2.3. Pre-processing

We used a custom implementation written in MATLAB (MathWorks, Natick, MA) and SPM5 (http://www.fil.ion.ucl.ac.uk/spm) to perform spatial preprocessing and calculation of the CBF-maps (see Preibisch et al., 2011 for details). In short, this included the following steps: (1) realignment of the time series of the control and tag images to correct for subject motion; (2) computation of 80 difference images from pairs of registered control and tag images; (3) computation of the average of the set of difference images; (4) segmentation and registration of gray matter (GM) and white matter (WM) probability maps to the difference images; (5) computation of the CBF (Nöth et al., 2006; Preibisch et al., 2011).

To avoid the misinterpretation of brain atrophy as CBF reduction, CBF values were corrected for partial volume effects (Johnson et al., 2005; Preibisch et al., 2011). After correction of CBF values, CBF maps were spatially normalized. Direct utilization of CBF maps was not possible in this step, since they show a low signal-to-noise ratio and few structural features. Therefore, we co-registered the mean of all tag and control images with the whole brain EPI reference image, which was aligned to the T1-weighted image. Then, we applied the same transformation to the CBF map. Finally, we spatially normalized T1-weighted structural images and applied the same transformation to CBF maps. The resulting CBF maps have a voxel size of 2 × 2 × 2 mm, given by the interpolation procedure.

### 2.4. Post-processing

The resulting CBF volumes are rather noisy because of the low volume fraction (~1–5%) of blood within tissue voxel. In order to reduce possible harmful effects of the noise, we performed a Gaussian smoothing procedure using different σs and kernel sizes *s*. We came to the conclusion that the best setting, i.e., providing the lowest classification error, was σ = 2 and *s* = 6.

Elderly subjects are more likely to be affected by cardiovascular deficiencies, which might lead to a globally reduced CBF. A globally reduced CBF, which is completely uncorrelated to the Alzheimer condition, might hamper the final diagnosis. Likewise, a systematic difference of labeling efficiency between scanners might influence global CBF. In order to mitigate the effects of age and related cardiovascular disorders as well as systematic differences in labeling efficiency, a feature scale normalization has been performed on each PASL volume. Motion analysis has been done to evaluate the rotational and translational movements of the participants. In order to avoid false results, five patients had to be excluded from the study due to their movements. These patients have already been excluded in the description of the subjects in Section 2.1.

In order to simplify the computation, every volume *I*_*n*_ has been linearized to the corresponding vector form Γ_*n*_, where *n* is the subject's index. The inverse linearization procedure is then applied to the results in order to obtain a volumetric image.

### 2.5. Model of the healthy population

In order to model the healthy population (i.e., reference model), an element-wise univariate Gaussian approach has been implemented by fitting a normal distribution in each voxel location. This is achieved by computing the two parameters of the distribution: mean μ and standard deviation σ (Equations 1a,b). This type of model is also known as moment driven, as the model is based on the moments required by the distribution. Leave-one-out cross-validation has been used during training. This model is used in a probabilistic setting. Nonetheless, the very same model is used for T-score estimation. The T-score estimate allows the voxel-wise comparison between a healthy model and a single subject. The final result provides an overview of the structural or functional abnormalities carried by an individual. We calculated the reference model with the healthy subjects (*n* = 32) and derived T-score maps for each individual subject for visual comparison.

(1a) $$\begin{array}{r} \Gamma_{\mu }=N_{HC}^{-1}\underset{N_{HC}}{\sum }\Gamma_{n}^{HC} \end{array}$$

(1b) $$\begin{array}{r} \Gamma_{\sigma }=N_{HC}^{-1}{\left(\underset{N_{HC}}{\sum }\left(\Gamma_{n}^{HC}-\Gamma_{\mu }\right)◦\left(\Gamma_{n}^{HC}-\Gamma_{\mu }\right)\right)}^{◦1∕2} \end{array}$$

where ◦ is the Hadamard product, Γ_μ_ is the element-wise mean, and Γ_σ_ is the element-wise standard deviation.

### 2.6. Voxel-wise comparison method

#### 2.6.1. Likelihood

In order to compare the model with an individual subject, we used the complementary cumulative Gaussian distribution and we computed the likelihood of deviant perfusion (Equation 2). The value represents a quantitative indicator of deviant perfusion: values close to 1 identify a severe hypo-perfusion, values close to 0.5 identify normal perfusion and values close to 0 identify hyper-perfusion.

(2) $$\begin{array}{l} L_{n}=1-\underset{t\;\leq \;\Gamma_{n}}{\sum }N_{◦}\left(t|\Gamma_{\mu },\Gamma_{\sigma }\right) \end{array}$$

where N_◦_ is the element-wise Gaussian density function.

The level of uncertainty in likelihood images is significant: any voxel-wise difference in the CBF with the normal population is classified as hypo- or hyper-perfused. In order to mitigate the effects of this uncertainty, we applied a prior probability distributions to the likelihood function (Equation 3, where *M* is the number of prior distributions).

(3) $$\begin{array}{l} P_{n}=M^{-1}\underset{M}{\sum }L_{n}◦\pi_{m} \end{array}$$

#### 2.6.2. First prior

An expert knowledge can be used as prior information. In this particular case, we know that values coming from the white matter are unreliable (Deibler et al., 2008). Thus, the amount of uncertainty can be reduced by removing non-gray matter signal via a masking procedure. A voxel-wise probabilistic mask of the gray matter (π_*GM*_) has been computed in the preprocessing step. This procedure also removed the non-brain signal induced by the smoothing procedure.

#### 2.6.3. Second prior

Different voxels might contribute differently to the final prediction. In order to quantify the contribution of each voxel location, ordinary least squares can be used (see Equation 4).

(4) $$\begin{array}{l} X\beta =\text{y}\;\text{where}\;\beta =\left(X^{-1}X\right)∕\left(X^{-1}y\right) \end{array}$$

Let the matrix *X* be the matrix of observed variables, built by stacking row vectors *L*_*n*_. The response vector y is a vector of dummy variables (i.e., each group is mapped to a numerical identifier): *HC* → −1, and *AD* → 1. Thus, the vector β represents the predictive contribution of each voxel location with respect to the disease. This system of equations is not directly solvable, as the number of features *J* is largely higher than the number of observed variables *N*. One way to overcome this problem is Principal Component Analysis (PCA).In particular, we applied a well-known PCA method for high-dimensional data (Bishop and Nasrabadi, 2007). This is done by (1) evaluating *XX*^*T*^ where *X* is the centered data matrix, (2) finding the eigenvalues and eigenvectors (λ_*j*_, v_*j*_) of *N*^−1^*XX*^*T*^ which are the same eigenvalues and eigenvectors of the original (but computationally infeasible) covariance matrix, and (3) computing the eigenvectors of the original data space using ${\text{u}}_{j}={\left(N\lambda_{j}\right)}^{-1∕2}X^{T}{\text{v}}_{j}$. Notice that we used only the first 20 components out of *N* available, as they were providing the minimum misclassification error. We reconstructed a reduced version of the original data matrix *X*′ = *XU* by projection. Once the dimensionality of the problem is reduced, we solved the linear system β′ = (*X*′ − 1*X*′) ∕ (*X*′ − 1y) and obtained the values of the reduced β. Once the vector of coefficients β′ is found, we projected it onto the principal components β = *Uβ*′ to obtain the full-dimensional approximation of β, which we used as second prior distribution π_*PP*_. Each π_*PP*_ obtained contains a value ranging from 0 to 1 which represents the predictive contribution of each voxel location.

This procedure resulted in a predictive map (**Figure 3D**) for each individual subject, which contains the values of the prior distribution ranging from 0 to 1. A leave-one-out strategy was used for training. Finally, we weighted the likelihood function with prior distributions to produce corrected posterior images *P*_*n*_ (Equation 3).

### 2.7. Discriminant analysis

Each resultant posterior volume contains measures of cortical perfusion whose deviations are correlated with the disease. Diseased patients are expected to have severe hypo-perfusion, or in other words, a high amount of voxels with low perfusion values, most likely within the parietal and temporal lobe.

In order to be able to discriminate each single volume between the two classes, we estimated the values of two thresholds: (1) the Within Subject Threshold (*t*_*w*_), which is the discriminant probability value to differentiate between a hypo-perfused voxel and a normally perfused voxel; and (2) the Between Subjects Threshold (*t*_*b*_): which is the number of voxels classified as hypo-perfused in a volume, which functions as partition hyperplane between the two classes. To determine both parameters we used a discrete evaluation of the accuracy response to determine the minimum misclassification rate. The corresponding sensitivity and specificity responses were also estimated. A leave-one-out strategy was used to compute the misclassification function producing a pair of parameter values in every iteration.

This type of discriminant analysis provides a simple, visual result. More advanced classification techniques can be used to improve the accuracy. Among the methods proposed in the machine learning literature, support vector machines (SVM) (Cortes and Vapnik, 1995) considered to be one of the most suited techniques to handle high dimensional spaces. The principle of SVM is to maximize the margin of the separation plane between two classes. Kernel functions, such as the radial basis function, can be used to handle non-linearly separable classes by projecting the data to a higher dimensional space. We applied SVM with radial basis kernels to the posterior probabilities returned by our method.

### 2.8. Regional analysis

In order to determine which brain regions were most affected by hypo-perfusion, we analyzed the cerebral distribution of hypo-perfused voxels using the Talairach brain atlas (Lancaster et al., 2000). This procedure was carried out by simple segmentation of each cerebral region for each subject, and accumulation of the total value for each region across the subjects. Prior to the segmentation procedure, every PASL volume has been co-registered in the same coordinate system of the Talairach brain template.

## 3. Results

Subjects characteristics along with biomedical and cognitive measurements are given in Table 1.

**Table 1:** **Clinical subject characteristics (cognitive and biomedical measurements) for each of the classification groups**.

**Category**	**Age**	**MMSE**	**Education (years)**	**Gender (Female / Male)**
*AD*_*LA*_	74.00 ± 6.70	21.93 ± 3.92	12.85 ± 3.95	15 / 15
*AD*_*EA*_	60.05 ± 6.38	22.35 ± 4.77	14.43 ± 4.50	10 / 9
*HC*	66.40 ± 6.19	29.46 ± 0.92	11.71 ± 3.24	22 / 10

The constructed reference model of normal cerebral blood perfusion, computed from the healthy group of subjects, is shown in **Figures 3A,B**. The mean (μ) and standard deviation (σ) of healthy controls is shown, where high blood perfusion (depicted in red) was mostly noticed in the parietal lobe and in the cuneus, visible in the left and right hemisphere view. For visual comparison, the CBF mean of the AD cohort is shown in **Figure 3C**. Although the perfusion in the AD cohort conserves a pattern similar to the one shown by the healthy perfusion, large cerebral areas in the parietal lobe are visibly less perfused compared to the mean of the healthy subjects (**Figure 3A**).

**Figure 2:**
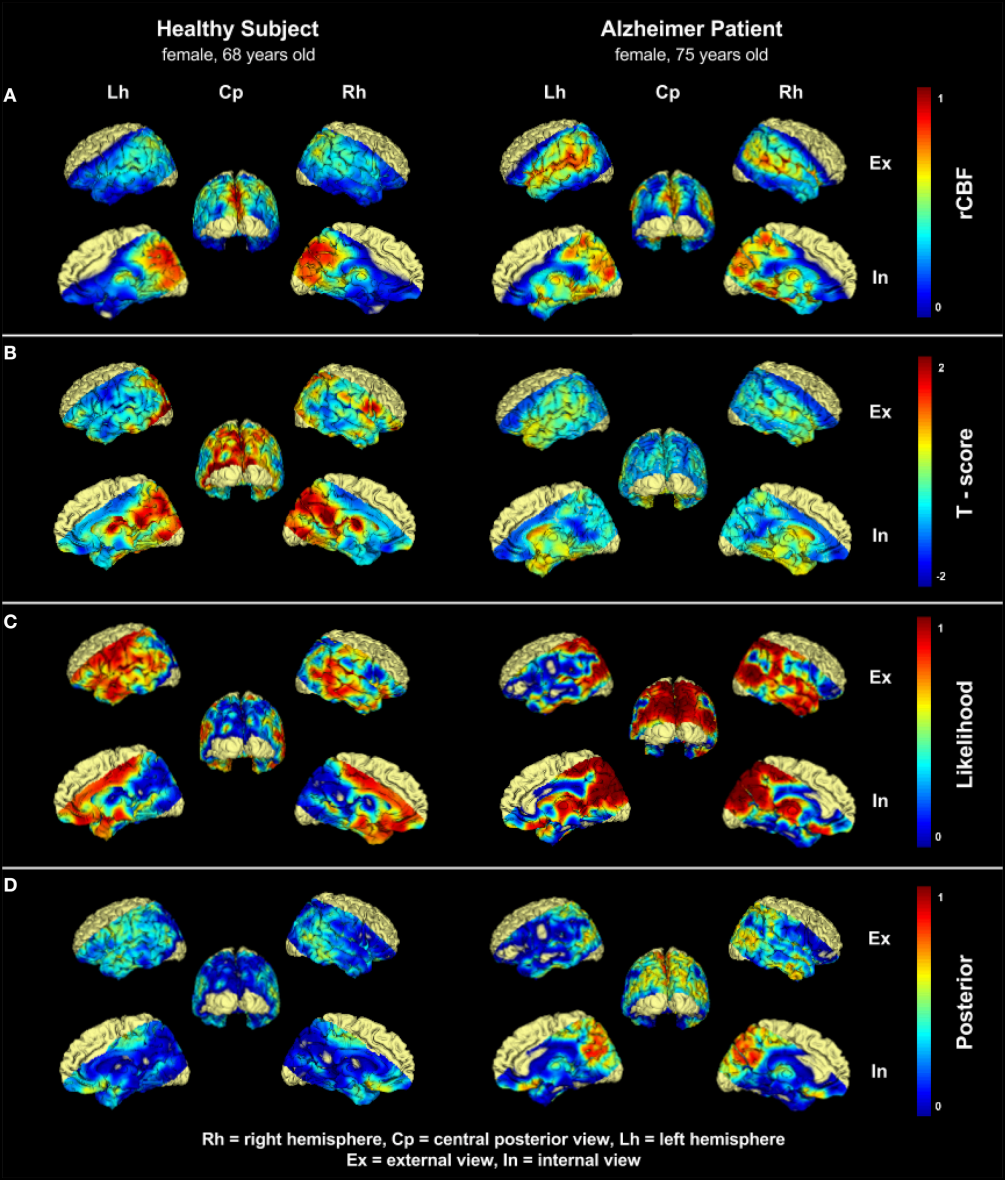
**Comparison of one 75 years old patient with Alzheimers disease (AD) and one 68 years old healthy control (HC)**. **(A)** shows the normalized CBF distribution, **(B)** shows the Tscore-map computed voxel-wise. A substantial difference could be already spotted in the parietal lobe. **(C)** shows the likelihood of hypo-perfusion L. In the AD patient severely hypo-perfused areas in the parietal lobe and neighboring regions are visible, whereas the HC subject shows regions with artificially low perfusion in the frontotemporal lobes and in the volume boundaries. These high values are the effect of uncertainty resulting from image noise, and/or patient-specific conditions. **(D)** shows the posterior probability (p), which represents the disease related likelihood of hypo-perfusion resulting from the application of the prior distribution. Areas with artificially low perfusion on volume boundaries have been suppressed, while the the sensitivity to interesting hypo-perfused region in the parietal lobe is still preserved. Upper labels Rh, Cp, and Lh indicates right hemisphere, central posterior, and left hemisphere, respectively. Lateral labels Ex and In indicates external view and internal view, respectively.

**Figure 3:**
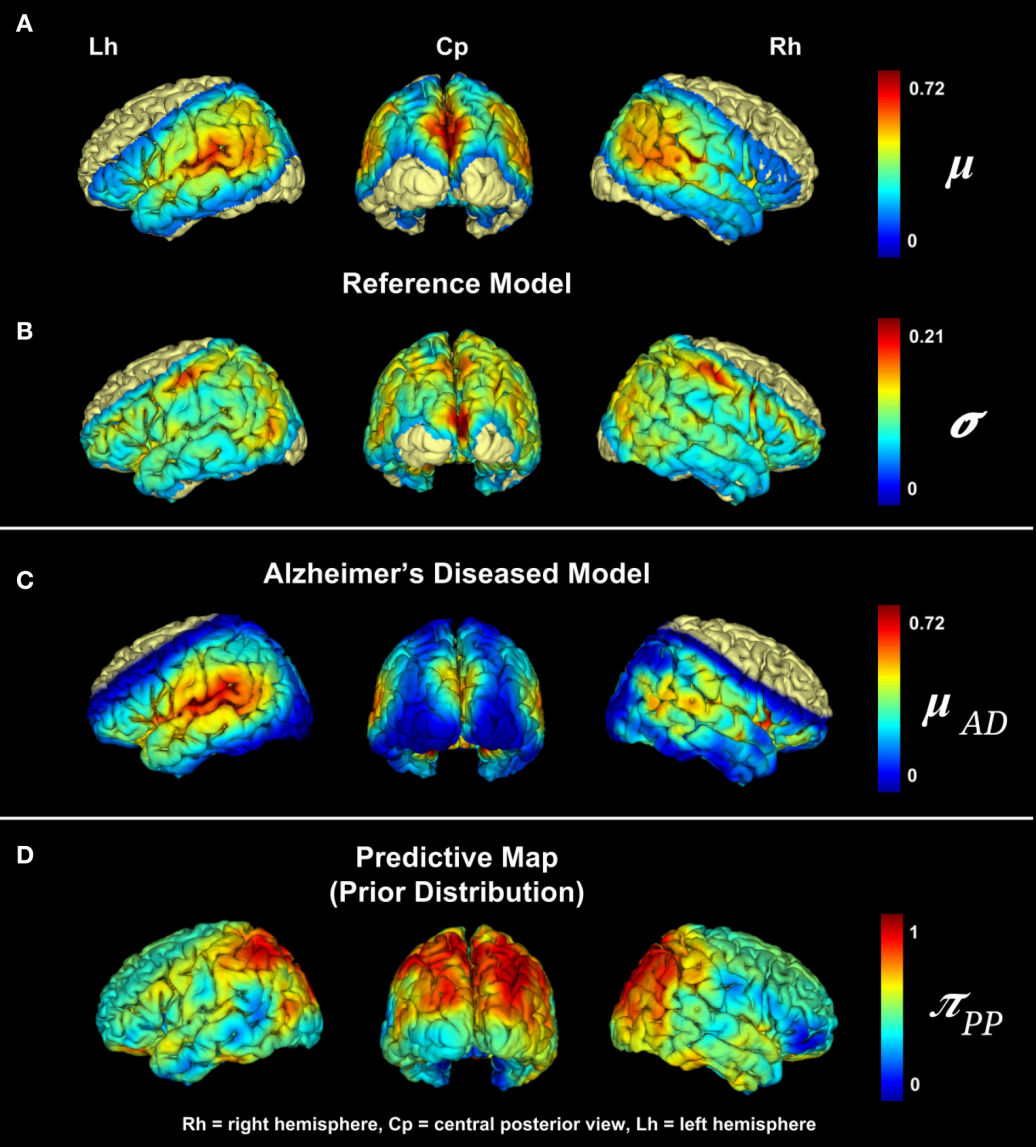
**Model of the cerebral blood perfusion based on HC subjects: (A) shows the mean and (B) the standard deviation of the healthy group**. High blood perfusion is visible in the parietal lobe and in the cuneus. We included for visual comparison **(C)**, which shows the mean perfusion of the AD cohort. **(D)** visualization of the prior distribution using principal component analysis to depict the predictive power of certain cerebral regions, where the parietal lobe showed the highest predictive power.

This observation is also confirmed by the prior distribution built upon a regression model (Figure 2D) where the parietal lobe and the surrounding regions evidenced a higher importance for the prediction of AD. Figure 2 shows a comparison between two randomly selected HC and AD subjects. Figures 2A,B represent the CBF and the T-score, respectively. Figure 2C shows the likelihood *L* using voxel-wise comparison. The healthy individual showed low perfusion in the frontotemporal lobe, which results from poor image quality due to susceptibility artifacts in these areas. We could reduce this effect of uncertainty in the likelihood images L by adding a prior distribution (Figure 3D). In the posterior images (*P*) the areas with low perfusion in healthy subjects have been suppressed, whereas regions of hypo-perfusion in the parietal lobes of AD subject were unaffected.

**Figure 4:**
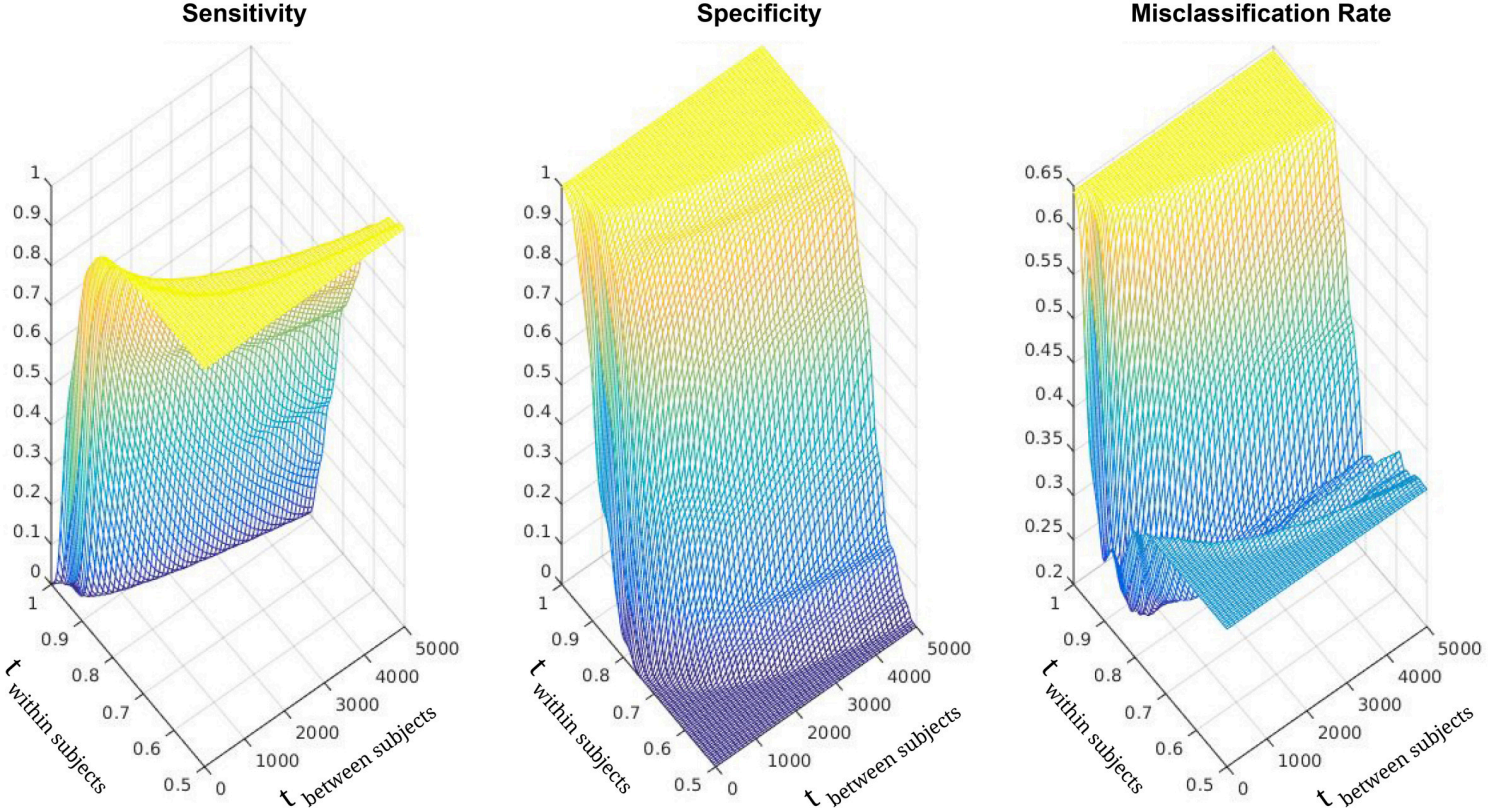
**Sensitivity, specificity, and misclassification rate functions**. Discrete representation over varying threshold values. The x-axis (ranging from 0 to 5000) is associated with the Between Subject Threshold. The y-axes (ranging from 1 to 0.5) is associated with the Within Subject Threshold.

For the discriminant analysis we determined the following thresholds: the within subject threshold *t*_*w*_, that is the probability for which a voxel shall be considered diseased, equals to 0.86; and the between subject threshold *t*_*b*_, which is the number of voxels for which a subject shall be considered diseased, equals to 200 (actually most of the runs in the testing phase produced *t*_*b*_ = 200 and *t*_*w*_ = 0.86). In Figure 4 specificity, sensitivity and misclassification functions for varying thresholds values are plotted.

Utilizing this method, we achieved a sensitivity of 0.750 and a specificity of 0.875 in the discrimination between healthy subjects (HC) and AD patients (*AD*_*EA*_, *AD*_*LA*_). Training the model with solely one subgroup of patients resulted in a sensitivity of 0.685 and specificity of 0.750 for the discrimination between HC subjects and *AD*_*EA*_ patients, and a sensitivity of 0.734 and specificity of 0.844 for the discrimination between HC subjects and *AD*_*LA*_ patients. The worsening of the classification quality is given by the reduced cohort of diseased patients, and the subsequent inability of the algorithm to properly model the disease. Finally, the application of SVM on the posterior probabilities outperformed the thresholds based classification: the resulting accuracy in the classification of HC vs AD was 0.9, with a sensitivity of 1 and a specificity of 0.75.

**Figure 5:**
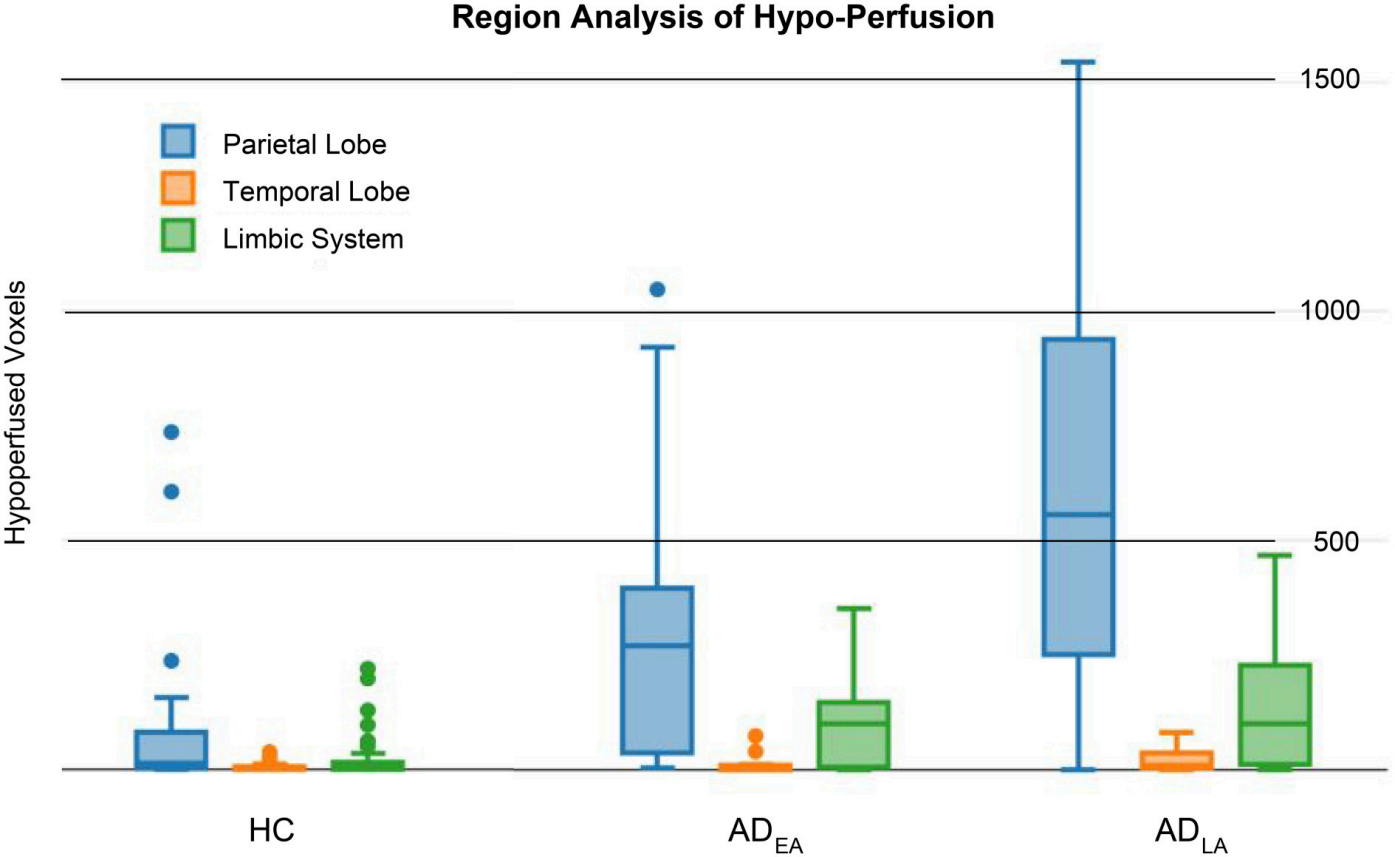
**Region Analysis**. Group distributions of hypo-perfused voxels in the parietal (blue), temporal (orange), and limbic system (green). Lobes which were not fully covered by the pASL signal have been excluded. Talairach brain atlas was used to perform the analysis.

With regional analysis we identified two distinct regions, which contain almost all hypo-perfused voxels: the parietal and temporal lobes, with particular focus on the limbic system, which on average accounted for 49 and 11% of the hypo-perfused voxels, respectively (Figure 5). We obtained significant differences in the parietal (*p* < 0.0001) and the limbic system (*p* = 0.0028).

The hypo-perfused volumes (i.e., the sum of hypo-perfused voxels) can also be correlated with clinical data available from each correctly classified patient. Particularly interesting is the high inverse correlation between the hypo-perfused volume and the MMSE score (ρ = −0.62, *p* < 0.001), which suggests the validity of the results. The weak correlation between age and hypo-perfusion (ρ = 0.32) can be explained by the characteristics of the cohort: healthy subjects tended to be slightly younger than patients. No correlation has been shown between hypo-perfusion and education (ρ = 0.09), which confirms the current state of the research (Burns and Iliffe, 2009; Kawas, 2012). APOE gene correlations have not been included, as data were available only for a few patients (see Table 1).

## 4. Discussion

In the present study, we investigated the diagnostic potential of PASL imaging in the diagnosis of AD. PASL offers two major advantages compared to other perfusion techniques. First, since PASL is an MR based method, the patient is not subjected to ionizing radiation. Second the method does not use any exogenous contrast agent and thus, is absolutely non-invasive. Thereby the risk of allergic reactions can be avoided and even patients with renal failure can be examined. This allows repeated and reproducible measurements which are important for follow-up studies. Several previous studies analyzed the CBF variations in patients with AD on a group level. As initial investigation in its diagnostic potential, we propose a method that provides both quantitative and visual results. Specifically, we extended VBM methodology, from a statistical tool modeling the differences between groups, to a quantitative and visual diagnostic procedure on a single subject level. This method encompasses the creation of a likelihood model and the refinement of the probabilities via prior distributions (i.e., predictive map) which account for the low signal-to-noise ratio and the signal-disease correlation. The comparison of the results of this method to other methods commonly used in the medical community is shown in Figure 2. Visual juxtaposition of rCBF maps (Figure 2A) or estimation of T-Score maps (Figure 2B) are affected by a non negligible amount of noise which makes it difficult to formulate a clear diagnosis. Likelihood maps on the other hand (Figure 2C), might already be capable of delineating some known characteristics of the disease, like the hypoperfusion located in the parietal lobe (Alsop et al., 2010). Nevertheless, the high variability of the results still does not allow a clear formulation of the diagnosis. The posterior distribution however (Figure 3D) provides a clear evidence of the disease by highlighting the voxel locations typically affected by disease related hypo-perfusion, and facilitating in this way the whole diagnostic process. Specifically, throughout the application of prior distributions, the likelihood values in areas with high gray matter probability and high disease-predictive power remain untouched. On the other hand, likelihood values in areas with low gray matter probability and/or low disease-predictive power will be down-weighted. This visual result is reflected by the quantitative analysis, and the high sensitivity and specificity. These outcomes have also been proven to be inline with the current pathological knowledge (Alsop et al., 2010): previous studies, in fact, reported hypo-perfusion in the precuneus and parietal cortex (parietal lobe), and the posterior cingulate (limbic system). In our results, we observed two cerebral regions responsible for most of the hypo-perfusion related to the disease: the parietal lobe and the limbic system. Minor differences have also been detected in the rest of the temporal lobe (see Figure 5). Furthermore, the correlation between cognitive impairment and hypo-perfusion volume suggests a possible link between them.

### 4.1. Limitations

The major limitation of this study is constituted by the cohort: the number of healthy subjects and *AD*_*EA*_ patients was relatively limited which limits the accuracy of the normal perfusion model. In addition to the size of the cohorts, also the age represents a limitation of this study, as the healthy subjects tended to be younger than diseased ones (*p* = 0.04) due to strict inclusion criteria. Finally, the utilization of two different MRI scanners, although both were using very similar settings, represents the last cohort-related limitation of this study. A larger cohort, especially with older healthy subjects and *AD*_*EA*_ subjects, whose PASL images have been acquired using the same scanner, might be beneficial. From a purely medical point of view, no subjects underwent examinations for the assessment of vascular diseases, such as stenosis. To this end, one might want to perform an analysis of the white matter lesions via Fazekas score or, even better, analyze extra- and intracranial vessels via doppler analysis or MR angiography to evaluate possible stenosis, which might have an influence on CBF maps. Further limitations are represented by the imaging modality: in order to obtain quantitative CBF values, a number of influences need to be considered (Deibler et al., 2008). For example, magnetically labeled blood decays with T1 of 1500 ms at 3.0T and as a result, sections acquired at last at the top of the volume will have less label than those acquired at the bottom. Moreover, the image acquisition process partially destroys the labeled spins and the transit time of the labeled blood to the imaging site plays an important role. In healthy subjects, the gray matter has a transit time that can vary between 500 and 1500 ms, whereas in deep white matter the transit time can be longer than 2000 ms (Alsop et al., 2015). In patients, there might even be longer delays due to vascular impairments which might result in an erroneous quantification of the CBF in the affected brain regions. Despite potential artifacts, the intrinsic non-invasiveness of PASL and the tight correlation between cerebral blood flow and cerebral activity make it a potentially attractive tool, worth to investigate its diagnostic capability in AD. Furthermore, PASL images are not covering the whole brain, but only a portion of it. Although the portion of the brain covered by the signal is actually fully covering those brain regions most affected by the disease, a full coverage could improve the diagnostic accuracy. Furthermore, as an MR based imaging technique, it encloses all contraindications of standard MRI, like the impossibility of usage for subjects with pacemakers and metal implants or claustrophobia.

### 4.2. Outlook

Recent pathophysiological studies (Verfaillie et al., 2015) also reported PASL imaging to effectively identify other types of dementia, like frontotemporal dementia. The application of diagnostic procedures in these cases should produce similar results as in Alzheimers dementia. Furthermore, better diagnostic results might be obtained using pseudo-Continuous Arterial Spin Labeling (pCASL), which is another MR based ASL imaging technique but, unlike PASL, is able to acquire perfusion images of the whole brain. Nonetheless, even if ASL is a promising technique, ASL still needs further evaluation in order to prove its clinical applicability. As significant variabilities can occur in the perfusion pattern especially in the early stages of the disease further examinations such as nuclear medicine techniques are still needed for diagnostics at this stage. Therefore, we are currently working on further analyze ASL, especially a comparison with PET.

### 4.3. Conclusion

This study presents initial results obtained with computer aided diagnostic (CAD) procedure for Arterial Spin Labeling (ASL) images in AD. In spite of suboptimal ASL data quality and brain coverage, this preliminary investigation showed good results and suggests that PASL might be actually suited to assist individual diagnosis of AD in the future, which principally opens a wide set of opportunities for an early, non-invasive diagnosis of AD.

## Author contributions

ST developed the method. IR, JK gave medical interpretations of the results. PA formulated the diagnosis. CP, SF, KB collected the data. AV, JK, CZ supervised the project.

### Conflict of interest statement

The authors declare that the research was conducted in the absence of any commercial or financial relationships that could be construed as a potential conflict of interest.
